# New index for distinguishing between critical illness myopathy and neuromyopathy: CMAP amplitude/duration index

**DOI:** 10.55730/1300-0144.5949

**Published:** 2024-12-30

**Authors:** Handan UZUNÇAKMAK UYANIK, Fatma Gökçem YILDIZ, Çağrı Mesut TEMUÇİN

**Affiliations:** 1 Institute of Neurological Sciences and Psychiatry, Hacettepe University, Ankara, Turkiye; 2 Department of Neurology, Faculty of Medicine, Hacettepe University, Ankara, Turkiye

**Keywords:** CMAP amplitude, critical illness myopathy, critical illness polyneuromyopathy, distal CMAP negative peak duration, index

## Abstract

**Background/aim:**

Critical illness-associated weakness is a common neuromuscular syndrome that may manifest as critical illness myopathy (CIM), critical illness polyneuropathy (CIP), or an overlapping syndrome known as critical illness polyneuromyopathy (CIPM). Distinguishing between these subtypes can be challenging due to technical issues, patient-related factors such as insufficient cooperation with needle electromyography (EMG) or edema, and the need for sophisticated, time-consuming electrophysiological methods and invasive procedures such as biopsy. This study aimed to contribute to the electrophysiological distinction between CIM and CIPM.

**Materials and methods:**

A new index was designed based on distal compound muscle action potential (CMAP) amplitude and negative peak duration values. Comparative and receiver operating characteristic curve analyses were performed on the parameters of patients with CIM and CIPM, as well as between patient groups and controls.

**Results:**

The median and ulnar CMAP index cut-off values for distinguishing between CIM and CIPM were determined to be 0.35 and 0.51, respectively. Values below these cut-off points support a diagnosis of CIPM while higher values indicate CIM. The best parameter for distinguishing both CIM and CIPM patients from healthy controls was the peroneal CMAP index, with a cut-off value of 0.45.

**Conclusion:**

The CMAP index can be easily calculated from CMAP values obtained during routine nerve conduction studies. This index may serve as a practical and guiding method for differentiating between CIM and CIPM, contributing to the electrophysiological diagnosis of critically ill patients, and particularly those with unreliable sensory nerve action potentials and needle EMG examinations.

## 1. Introduction

Critical illness-associated weakness (CIAW), a common neuromuscular syndrome, may manifest as critical illness myopathy (CIM), critical illness polyneuropathy (CIP), or an overlapping syndrome known as critical illness polyneuromyopathy (CIPM) [1]. The incidence of CIAW has been reported to range from 25% to 31% [1–5]. Common causes of CIAW include sepsis, multiorgan failure, mobility restriction, lung diseases, hyperglycemia, the use of glucocorticoids, and neuromuscular blocking agents [6]. The typical presentation of CIAW is symmetrical, with flaccid limb weakness that more severely affects proximal limb muscles [7] and difficulty in weaning the patient from the ventilator [1].

There is ongoing debate regarding which subtype of CIAW is more common. Many publications suggest that CIPM is the most common manifestation of CIAW [8–11]. Additionally, it has been proposed that the prognosis for CIM is better than that for CIP [12,13]. Distinguishing between these subtypes is important due to their different prognoses. The methods used to diagnose CIAW include manual muscle testing (MMT), electromyography (EMG), and muscle or nerve tissue pathology [1]. However, since only 25%–29% of patients are conscious during these examinations, the contribution of MMT to diagnosis is limited, and biopsy is not preferred due to its invasive nature and potential complications.

Currently, noninvasive electrophysiological tests for CIAW have the highest diagnostic value, with findings detectable 24–48 h after the onset of pathology and before clinical symptoms appear [14]. In this context, sensory nerve conduction studies (NCSs) can be crucial in distinguishing between CIM and CIP. However, cold extremities, edema, and artifacts in the intensive care unit (ICU) environment may hinder the accurate acquisition of sensory nerve action potentials (SNAPs). Furthermore, distinguishing between myopathy and neuropathy can be problematic in ICU patients due to severe weakness, impaired voluntary effort, or altered mental status. Lastly, both conditions can present with low compound muscle action potential (CMAP) amplitudes, prolonged distal CMAP negative peak durations, and synchronous dispersion of proximal and distal CMAPs as specific features suggesting the presence of CIM [15,16]. Thus, we aimed to contribute to the electrophysiological distinction between CIM and CIPM by designing a new index based on distal CMAP amplitudes and negative peak durations.

## 2. Materials and methods

Our EMG laboratory’s database was reviewed retrospectively from July 7, 2017, to March 16, 2023, using the keywords “critical illness myopathy,” “critical illness polyneuropathy,” “critical illness polyneuromyopathy,” “intensive care polyneuropathy,” “intensive care myopathy,” and “intensive care polyneuromyopathy.” Patients referred to the EMG laboratory with clinical findings of CIAW and a confirmed electrophysiological diagnosis of critical illness myopathy/neuropathy/polyneuropathy were included in this study. All sociodemographic, clinical, and laboratory data of the patients were extracted from the medical recording system. A total of 154 nerves from 30 healthy participants with normal routine NCSs were included in the study as the control group. Approval for the study was granted by the Ethics Committee of the Hacettepe University Ethics Board (No. GO 23/426).

NCS recordings were amplified, filtered, and stored using a Keypoint EMG machine (Alpine Biomed, Skovlunde, Denmark). Filter settings were 20 Hz to 2 kHz and 20 Hz to 10 kHz for sensory and motor conduction study recordings, respectively. The NCS examination included sensory and motor NCS performed on the right side using surface silver/silver chloride disc recording electrodes. Standard motor NCSs included peroneal motor NCS recordings at the extensor digitorum brevis muscle, tibial motor NCS recordings at the abductor hallucis, median motor NCS recordings at the abductor pollicis brevis, and ulnar motor NCS recordings at the abductor digiti minimi using a belly-tendon montage. Sensory NCSs included orthodromic ulnar and antidromic sural sensory nerve studies. Notably, since the presence of sural SNAPs and their amplitudes (if present) is critical for distinguishing neuropathy, the antidromic method was preferred while avoiding stimulating the motor fibers. Additionally, the skin temperature was maintained at or above 32 °C.

The following variables were evaluated: the amplitude of the compound muscle action potential (CMAP_amplitude_), the distal CMAP negative peak duration (CMAP_duration_), and the amplitude of the sensory nerve action potential (SNAP_amplitude_). CMAP_amplitude_ was measured from the baseline to the negative peak. CMAP_duration_ was defined as the time from baseline to the negative peak. NCS results, including CMAP_duration_, were evaluated according to the normal ranges of our laboratory. Additionally, CMAP_amplitude_/CMAP_duration_ index values were calculated for the median, ulnar, peroneal, and tibial motor nerves.

Data analyses were performed using IBM SPSS Statistics 26.0 (IBM Corp., Armonk, NY, USA). For categorical variables, numbers and percentages were reported; for continuous variables, mean ± standard deviation or median and range (minimum–maximum) values were used. Chi-square tests were used for intergroup comparisons of categorical variables. The normality of continuous variables was assessed using Kolmogorov–Smirnov and Shapiro–Wilk tests. As the parameters did not show a normal distribution, the Kruskal–Wallis test was employed to compare the CMAP_amplitude_, CMAP_duration_, CMAP_amplitude_/CMAP_duration_, and SNAP_amplitude_ parameters across the groups, while the Mann–Whitney U test was used for pairwise group comparisons. Holm correction was applied. Significance was established at p < 0.05.

To estimate the best cut-off value for distinguishing between CIM and CIPM patients based on the CMAP_amplitude_/CMAP_duration_ index, receiver operating characteristic (ROC) curve analysis was performed and the area under the curve (AUC) was calculated. When a significant cut-off value was observed, sensitivity and specificity values were presented. The outcomes were reported as AUC, cut-off criterion, sensitivity, and specificity values. A p-value of less than 0.05 was considered statistically significant. To enhance specificity and sensitivity, ROC analysis was conducted using multiple parameters combined with logistic regression. Additionally, to differentiate between patients and control group participants, cut-off values for the ulnar, median, peroneal, and tibial CMAP_duration_, as well as for the CMAP_amplitude_/CMAP_duration_ index, were also determined using ROC analysis.

## 3. Results

### 3.1. Demographic and clinical features

Sixty-six patients with probable critical illness myopathy and/or polyneuropathy according to relevant diagnostic criteria were included in this study [13]. EMG was performed within the first 4 days of symptom onset. Of the 66 patients, 32 (48.5%) were female. The mean age of these patients was 57.97 ± 19.27 years and the median age was 62 (range: 17–88).

We classified the causes of hospitalization into five main categories: sepsis, trauma, neurological diseases, postoperative complications, and others. The “others” category included complications related to malignancy, lung diseases, attempted suicide, metabolic acidosis, and hemophagocytic syndrome.

**Figure 1:**
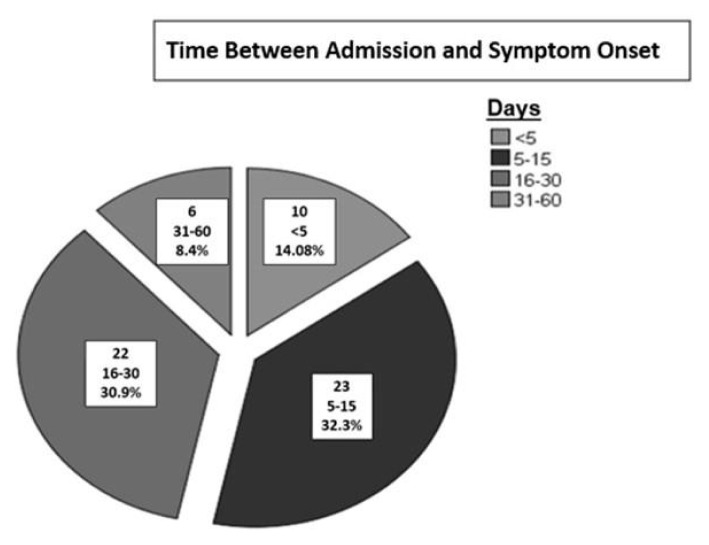
Time between admission and symptom onset.

Of the 66 patients, 45 (68.1%) were diagnosed with CIPM, 19 (28.7%) with CIM, and 2 (3%) with CIP. The most common cause of hospitalization for both CIM and CIPM patients was infection/sepsis (Table 1). The most prevalent comorbidity among the patients was diabetes mellitus (n=14, 21.2%) (Table 1). The time between admission and symptom onset, which could be determined from database notes, was evaluated. The duration between admission and detection of weakness and/or difficulty in extubation ranged from 1 to 60 days, with the highest frequency occurring between 5 and 15 days among all patients (32.3%) (Figure 1). The number of patients who tested positive for COVID-19 at the time of diagnosis was 3 in the CIM group and 5 in the CIPM group.

Statistical analyses were performed with the data of CIM and CIPM patients, as there were only 2 CIP patients. There was no significant difference between the two groups in terms of sex (p = 0.21), age (p = 0.54), COVID-19 positivity (p = 0.3), time between admission and symptoms (p = 0.32), or creatine kinase (CK) levels (p = 0.83). Additionally, while no statistically significant difference was found between the patient and control groups in terms of sex (p = 0.379), a significant difference was identified in terms of age (p = 0.016).

### 3.2. Electrophysiological data

Table 2 includes the median, minimum, and maximum values of the CMAP_amplitude_, CMAP_duration_, CMAP_amplitude_/CMAP_duration_, and SNAP_amplitude_ parameters for all nerves. Kruskal–Wallis tests showed significant differences between the groups (Table 2). Post hoc analyses revealed significant differences in the median, ulnar, peroneal, and tibial nerve CMAP_amplitude_, CMAP_duration_, CMAP_amplitude_/CMAP_duration_, and sural nerve SNAP_amplitude_ parameters between the control group and both the CIM and CIPM groups. Although there were no significant differences in median, ulnar, peroneal, and tibial nerve CMAP_duration_ parameters between the CIM and CIPM groups, the differences among the median, ulnar, and tibial nerve CMAP_amplitude_/CMAP_duration_ parameters were significant. There were no significant differences for the peroneal nerve CMAP_amplitude_/CMAP_duration_ parameter between the CIM and CIPM groups.

**Figure 2:**
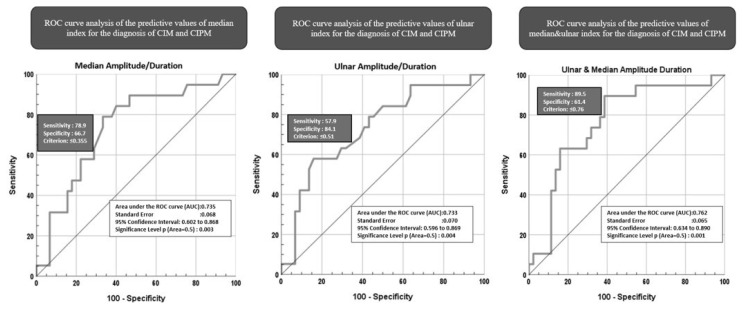
ROC curve analyses of patients. CIM: Critical illness myopathy; CIPM: critical illness polyneuromyopathy.

In ROC analysis, the cut-off value for the peroneal CMAP_amplitude_/CMAP_duration_ index was determined to be 0.45 (with an AUC of 1) for our patient and control groups. The median nerve CMAP_amplitude_/CMAP_duration_ index for distinguishing between CIM and CIPM exhibited 78.9% sensitivity and 66.7% specificity, with an AUC of 0.735. The cut-off was determined to be ≤0.35 (Figure 2). Values lower than this cut-off support a CIPM diagnosis, while higher values support a CIM diagnosis. The ulnar nerve CMAP_amplitude_/CMAP_duration_ index for distinguishing between CIM and CIPM had 57.9% sensitivity and 84.1% specificity, yielding an AUC of 0.733. The cut-off was determined as ≤0.51 (Figure 2). To achieve higher specificity and sensitivity, ROC analysis was conducted using median and ulnar CMAP_amplitude_/CMAP_duration_ values in a model combined with logistic regression. The Youden index value was found to be 0.762 (Figure 2); however, the combined parameter index did not demonstrate superiority over the indices of the individual parameters.

ROC analysis was also conducted using CMAP_amplitude_ to distinguish between CIM and CIPM. The sensitivity and specificity were 68.4% and 68.2% for the CMAP_amplitude_ values of both the ulnar and median nerves. The AUC was 0.754 for the ulnar CMAP_amplitude_ and 0.737 for the median nerve CMAP_amplitude_. Although the AUCs for both the CMAP_amplitude_ and index analyses were close to each other, the sensitivity of median CMAP_amplitude_ and the specificity of ulnar CMAP_amplitude_ did not achieve the sensitivity of the median index or the specificity of the ulnar index. The CMAP_amplitude_/CMAP_duration_ of the tibial and peroneal nerves were not useful in differentiating CIM from CIPM due to low sensitivity and specificity at acceptable AUCs for the cut-off value.

## 4. Discussion

The frequency of each subtype of CIAW is a subject of debate, which may be partly related to the challenges in distinguishing between CIM, CIP, and CIPM. To address this issue, we aimed to define a new index based on distal CMAP amplitude and negative peak duration parameters obtained during routine NCSs. As the study included a small sample of CIP patients, we focused on distinguishing between CIM and CIPM.

Neuropathic involvement in the form of CIP or CIPM is typically characterized as symmetrical, length-dependent sensory-motor axonal polyneuropathy. Many prospective studies have revealed that 47% to 70% of critically ill patients develop electrophysiological evidence of this condition, usually within 1–3 weeks, which is consistent with our findings [17,18]. However, although pure sensory and motor neuropathy have been reported [8,19], the existence of these forms remains somewhat controversial. Koch et al. diagnosed 68% of patients with CIM and 38% with CIP [10]. Crone reported four patients with CIP in a study of 45 patients [20]. Our study identified only two patients with CIP (2.8%).

Given that neuropathic involvement in CIAW has a worse prognosis in terms of both neuropathic pain and motor recovery [21], distinctions between CIP, CIPM, and CIM are critical for prognosis prediction. These distinctions may also be of importance regarding physical therapy applications. Although some promising biomarkers have been reported for early diagnosis, such as C-terminal agrin, interleukin-6, and growth-differentiation factor-15, these are not yet routinely utilized [1]. CK levels are usually mildly and transiently elevated in CIAW; however, normal values do not rule out myopathy. Significantly high values are often seen in the necrotizing variant [22]. Bednarik et al. detected elevated CK values in 35% of patients with CIPM [9]. In our study, CK levels were normal in 24 (33.8%) of 66 patients but were >1000 U/L in 12 patients (16.9%).

Moreover, biopsy has limitations for diagnosis due to its invasive nature. In one study, CIP was ascertained by EMG in 92% of CIAW patients, whereas only 36% had abnormalities in nerve biopsy [8]. Additionally, muscle biopsy showed histological myopathic changes in 79% of the patients [8]. These results were attributed to functional changes that appear earlier than structural changes.

Given these challenges, the significance of EMG methods can be highlighted once again. The difficulties in diagnosis have directed EMG specialists toward sophisticated methods such as direct muscle stimulation (DMS) and motor unit number estimation (MUNE) as well as practical EMG techniques. However, methods such as DMS and MUNE, which indicate reduced excitability, are often complex and time-consuming, making them impractical for routine use and requiring considerable expertise. Furthermore, Seghelini highlighted that discrepancies may arise between the results of DMS and muscle biopsy [23].

Previously, a multicenter study stated that a peroneal CMAP amplitude reduction below 2 standard deviations of the normal value could be used as a screening criterion for CIAW, though it could not differentiate between subtypes, and the presence of low SNAPs was attributed to coexisting neuropathy or a technical issue [24]. That study further indicated that if the CMAP amplitude reduction is disproportionate to the corresponding SNAP amplitude reduction, it is suggestive of CIM (e.g., a median CMAP amplitude of 1 mV and a median SNAP amplitude of 10 μV) [24].

In our study, we found a significant difference in CMAP_amplitude_ values between the patient groups. To the best of our knowledge, no other studies have documented lower amplitudes in CIPM compared to CIM, as we observed. However, it is important to recognize that in the CIPM group, the low amplitudes cannot be solely attributed to CIAW, as various etiologies, such as diabetes mellitus and malignancy, can also lead to axonal neuropathy. Additionally, considering that polyneuropathy is common in the general population, it is possible that this condition preexisted in some patients. Moreover, given that baseline EMG results are often unavailable, any preexisting neuropathy may be incorrectly attributed to new developments arising from critical illness. Therefore, it is essential to establish more reliable measurements to define CIM accurately and differentiate it from CIP and CIPM.

Following its initial description in the early 2000s [25,26], prolonged CMAP duration and synchronous dispersion, unlike the asynchronous dispersion seen in demyelinating pathologies, was suggested as a characteristic feature of CIM [26,27]. In CIM, CMAPs evoked by the stimulation of motor nerves exhibit reduced amplitudes and increased CMAP durations with synchronous dispersion. A significant difference in CMAP_duration_ was observed for all nerves between the CIM and control groups. Therefore, the prolongation in CMAP_duration_, which is claimed to be quite specific for CIM, may also be useful in distinguishing between CIM and CIPM. However, in our study, no statistically significant differences were observed in CMAP_duration_ between these two groups for any nerve.

In light of these findings, it is essential to establish more reliable measurements to define CIP and CIPM accurately and differentiate them from CIM. In axonal neuropathies, a prolongation of CMAP_duration_ can be unexpectedly observed alongside a decrease in CMAP_amplitude_. Furthermore, in CIM and CIPM, CMAP_duration_ may sometimes reach the upper limit of normal, despite disproportionately low CMAP_amplitude_ [28]. These findings observed in CIAW patients may be mistakenly attributed to CIM instead of neuropathy.

Considering the low amplitude of CMAP and the prolongation or preservation of its duration, we developed the CMAP_amplitude_/CMAP_duration_ index based on these two CMAP parameters. We hypothesized that, in conditions such as CIM and CIPM, this index could reflect and quantify the changes between CMAP amplitude and duration, which occur in the opposite directions. Even in the absence of significant prolongation, a relatively preserved distal CMAP negative peak duration could indicate important changes; thus, detection of these variations through the CMAP_amplitude_/CMAP_duration_ index could serve as a valuable clue for diagnosing CIM and distinguishing between CIM and CIPM. In our study, the CMAP_amplitude_/CMAP_duration_ index values showed significant differences between the two patient groups (CIM and CIPM), with the exception of the peroneal nerve. The longest CMAP duration in CIM was detected for the peroneal nerve, recorded over the tibialis anterior (>15 ms) [29]. Generally, for the practical diagnosis of patients with CIM and CIP, the peroneal nerve has been selected from among the motor nerves in most studies [24,30–32]. In these studies, it was stated that the peroneal nerve was the motor nerve with the highest sensitivity in distinguishing between patients and controls. Meldgaard et al. evaluated muscle velocity recovery cycles in the tibialis anterior muscle in the early diagnosis of CIM [33]. Considering these findings, it can be thought that the peroneal CMAP is the CMAP most prone to be involved in both CIM and CIPM; thus, it is nonspecific in distinguishing between CIM and CIPM. Therefore, the lack of a significant difference in the peroneal nerve index in the distinction between CIM and CIPM may not be surprising.

The index values were significantly lower in the CIM and CIPM groups compared to the control group in this study, as CMAP_amplitude_ was decreased while CMAP_duration_ was prolonged or relatively preserved due to myopathy. This index appears to be a robust tool for distinguishing CIM and CIPM from a control group.

The CMAP_amplitude_/CMAP_duration_ index was also significantly lower in the CIPM group compared to the CIM group. In addition to the prolonged or relatively preserved CMAP_duration_ values due to myopathy in both groups, we suggest that this finding is related to the axonal involvement in CIPM, which leads to an additional reduction in CMAP_amplitude_ in CIPM compared to CIM.

ROC analysis of the CMAP_amplitude_/CMAP_duration_ index provided cut-off values (0.35 for the median nerve and 0.51 for the ulnar nerve) that could be used to distinguish the two patient groups. However, the percentages obtained in ROC analysis using CMAP_amplitude_ to differentiate between CIM and CIPM did not achieve the sensitivity of the median index or the specificity of the ulnar index.

Although we lacked an axonal neuropathy group comprising etiologies other than CIPM for testing since there would be no accompanying prolongation in CMAP duration, we anticipate that the index values in other axonal neuropathy groups will not be as low as those found in CIPM. Further studies that include axonal neuropathy groups other than CIPM may provide objective data on this issue.

Our results showed that both median and ulnar nerve CMAP_amplitude_/CMAP_duration_ indices are useful for distinguishing between CIM and CIPM, while the tibial index is not. This is likely due to the following factors: the p-value for the tibial index was closer to 0.05 compared to the ulnar and median indices, our study included fewer CIM patients, and we had overlapping values between the CIM and CIPM groups. Notably, in light of the existing literature, we had predicted that the peroneal CMAP_amplitude_/CMAP_duration_ index would exhibit the highest sensitivity and specificity among motor nerves. However, contrary to the literature, the median and ulnar nerve indices demonstrated higher sensitivity and specificity for differentiation [15,30]. Notwithstanding the modest sensitivity and specificity, likely due to the smaller sample size, we speculate that the CMAP_amplitude_/CMAP_duration_ index could provide useful insights for distinguishing between CIM and CIPM.

The retrospective nature of our study, the small sample of CIP patients, the relatively small number of CIM patients, and the absence of an axonal neuropathy group other than CIPM for testing are considered limitations of this study.

In conclusion, to evaluate the relationships between CMAP amplitude and duration in distinguishing between CIPM and CIM, we conducted a retrospective clinical and electrophysiological study involving critically ill patients. We believe that this index may serve as a practical and valuable tool for differentiating between CIM and CIPM, thereby improving electrophysiological diagnoses for critically ill patients and particularly those with unreliable SNAPs and needle EMG examinations.

## Figures and Tables

**Table 1:** Demographic features of participants and clinical characteristics of patients. Tables were prepared using data obtained from the laboratory’s database.

Demographic Features of Participants										
			Patients (Number of female/total = 32/66)							Controls (n = 30)
			CIM (n = 19)			CIPM (n = 45)	CIP (n = 2)			
**Mean age (SD)**			50.7(20.5)			61(18.4)	57.5(12)			48.1(15.2)
**Number of female(%)**			11(%57.8)			21(%46.6)	0			23(%76.6)
**Cause of Hospitalization (% within diagnosis)**										
	**n**	Number of patients with no data available	**Sepsis**		**Neurological disease**	**Trauma**	**Postoperative**			**Others**
**CIM**	19	2	8 (47%)		1 (5.8%)	-	1 (5.8%)			7 (41.1%)
**CIPM**	45	15	20 (66.6%)		8 (26.6%)	1 (3.3%)	1 (3.3%)			-
**CIP**	2	**-**	**-**		1 (50%)	1 (50%)	-			-
**CK Levels (% within diagnosis)**										
	**n**	Number of patients with no data available	**<145 U/L**		**146–300 U/L**	**301–1000 U/L**	**1001–10,000 U/L**			**>10,000 U/L**
**CIM**	19	1	7 (38.8%)		3 (16.6 %)	5 (27.7 %)	3 (16.6 %)			-
**CIPM**	45	10	15 (42.8%)		5 (14.2 %)	6 (17.1 %)	8 (22.8 %)			1 (2.8 %)
**CIP**	2	1	1 (100%)		-	-	-			-
**Comorbidities (% within diagnosis)**										
	**n**	Number of patients with no data available	Diabetes Mellitus	Malignancy	Lung disease (non-malign)	Neurological disease	Rheumatic disease	Hypertension	Renal disease (nonmalign)	Cardiac disease
**CIM**	19	1	3 (16.6%)	5 (27.7%)	4 (22.2%)	4 (22.2%)	3 (16.6%)	2 (11.1%)	-	2 (11.1%)
**CIPM**	45	3	9 (21.4%)	13 (30.9%)	3 (7.1%)	2 (4.7%)	1 (2.3%)	8 (19%)	2 (4.7 %)	4 (9.5 %)
**CIP**	2	-	2 (100%)	-	-	-	-	-	-	-

CIM: Critical illness myopathy; CIPM: critical illness polyneuromyopathy; CIP: critical illness polyneuropathy; CK: creatine kinase.

**Table 2:** Electrophysiological findings and comparative analysis results of patients and controls.

	Ulnar Motor Nerve			Median Motor Nerve			Peroneal Motor Nerve			Tibial Motor Nerve			Sural Nerve
	CMAP_amplitude_ (mV)	CMAP_duration_ (ms)	CMAP_amplitude_/CMAP_duration_	CMAP_amplitude_ (mV)	CMAP_duration_ (ms)	CMAP_amplitude_/CMAP_duration_	CMAP_amplitude_ (mV)	CMAP_duration_ (ms)	CMAP_amplitude_/CMAP_duration_	CMAP_amplitude_ (mV)	CMAP_duration_ (ms)	CMAP_amplitude_/ CMAP_duration_	SNAP amplitude (μV)
**Control**	8.55 (5.7***–***12.3)	4.7 (3.9***–***5.6)	1.76 (1.16***–***2.93)	8 (6.2***–***13.4)	4.8 (3.8***–***6.6)	1.72 (1.05***–***2.88)	4.7 (2.8***–***7.7)	5.4 (4***–***7.4)	0.89 (0.51***–***1.71)	10.4 (5.6***–***19.7)	5.6 (3.2***–***6.8)	1.89 (1***–***4.24)	15.4 (8.1***–***39.7)
Median (Min-Max)													
**CIM**	3.8 (0.5***–***7.2)	6.7 (4.3***–***11.7)	0.57 (0.06***–***1.67)	4.3 (0.2***–***9.7)	6.3 (4.6***–***8.9)	0.51 (0.06***–***1.28)	0.6 (0***–***2.9)	7.7 (5.1***–***14)	0.09 (0.02***–***0.4)	12.6 (3.9***–***21.5)	7.2 (4.2***–***10.9)	0.55 (0.02***–***1.7)	10.3 (4***–***21.5)
Median (Min-Max)													
**CIPM**	2.2 (0***–***7.6)	7.3 (4.4***–***11.3)	0.29 (0.02***–***1.25)	1.6 (0***–***8)	7 (4.6***–***11.2)	0.29 (0.01***–***1.2)	0.1 (0***–***2.6)	7.5 (4.2***–***15.2)	0.07 (0.01***–***0.4)	3.7 (0***–***8.3)	6.6 (4***–***17)	0.23 (0.01***–***1.36)	2.42 (0.04***–***6)
Median (Min-Max)													
**Kruskal Wallis Test**													
**χ2**	62.388	53.443	62.661	64.849	42.612	63.352	65.033	28.438	51.206	61.783	24.154	58.828	66.053
**df**	2	2	2	2	2	2	2	2	2	2	2	2	2
**p value**	**0.000**	**0.000**	**0.000**	**0.000**	**0.000**	**0.000**	**0.000**	**0.000**	**0.000**	**0.000**	**0.000**	**0.000**	**0.000**
**Post-hoc Pairwise Comparisons with Mann Whitney U Test (p value)** **													
**CIM vs. control**	**0.000**	**0.000**	**0.000**	**0.000**	**0.000**	**0.000**	**0.000**	**0.000**	**0.000**	**0.000**	**0.000**	**0.000**	**0.012**
**CIPM vs. control**	**0.000**	**0.000**	**0.000**	**0.000**	**0.000**	**0.000**	**0.000**	**0.000**	**0.000**	**0.000**	**0.000**	**0.000**	**0.000**
**CIM vs. CIPM**	**0.003**	0.130	**0.004**	**0.003**	0.176	**0.003**	**0.001**	0.445	0.258	**0.002**	0.679	**0.026**	**0.000**

CIM: Critical illness myopathy; CIPM: critical illness polyneuromyopathy; Min: minimum; Max: maximum; CMAP: compound muscle action potential; SNAP: sensory nerve action potential; CMAP_duration_: distal compound muscle action potential negative peak duration; CMAP_amplitude_/CMAP_duration_: distal CMAP amplitude/negative peak duration;

** Holm correction was applied.
